# Plastisch-chirurgische Therapie der Neurofibromatose Typ 1

**DOI:** 10.1007/s00104-024-02232-5

**Published:** 2025-01-28

**Authors:** Gregor Längle, Andreas Gohritz, Clemens Gstöttner, Leopold Harnoncourt, Hannes Platzgummer, Amedeo A. Azizi, Oskar Aszmann

**Affiliations:** 1 Universitätsklinik für Plastische, Rekonstruktive und Ästhetische Chirurgie, Medizinische Universität Wien, Wien, Österreich; 2 Plastische, Rekonstruktive und Ästhetische Chirurgie, Handchirurgie, Universitätsspital, Basel, Schweiz; 3 Klinische Abteilung für Neuroradiologie und Muskuloskeletale Radiologie, Universitätsklinik für Radiologie und Nuklearmedizin, Medizinische Universität Wien, Wien, Österreich; 4 Abteilung für Neonatologie, Pädiatrische Intensivmedizin und Neuropädiatrie, Universitätsklinik für Kinder- und Jugendheilkunde, Medizinische Universität Wien, Wien, Österreich; 5 Europäisches Referenznetzwerk für Genetische Tumorrisikosyndrome (ERN GENTURIS), Nijmegen, Niederlande

**Keywords:** Neurofibrom, Pathogenese, Diagnostik, Plastische Chirurgie, Maligne periphere Nervenscheidentumoren, Neurofibroma, Pathogenesis, Diagnostics, Plastic surgery, Malignant peripheral nerve sheath tumors

## Abstract

Die Neurofibromatose Typ 1 (NF1, früher Morbus Recklinghausen) ist ein genetisches Tumorprädispositionssyndrom, bei dem die Mutation eines Tumorsuppressorgens (Neurofibromin) zur Ausbildung meist gutartiger Neurofibrome der Haut und des zentralen und peripheren Nervensystems und Fehlbildungen oder Tumoren anderer Organsysteme führt. Patient:innen mit NF1 sollten lebenslang in spezialisierten Zentren interdisziplinär betreut werden, wichtige Therapieentscheidungen sollten hier in einem regelmäßig stattfindenden interdisziplinären Expertengremium getroffen werden. Die plastische Chirurgie spielt in der Behandlung aller klinischen Formen der NF1-assoziierten Nervenscheidentumoren eine wichtige Rolle – von kutanen und subkutanen bis zu tiefen nodulären und diffusen plexiformen Neurofibromen. Jeder Patient erfordert eine individualisierte chirurgische Planung, wobei Zeitpunkt und Ausmaß der Operationen von Symptomatik, funktionellen und ästhetischen Einschränkungen, Fortschreiten der Krankheit und potenzieller maligner Entartung bestimmt werden. Da jede Körperregion betroffen sein kann, reicht das erforderliche umfassende Spektrum ästhetischer und rekonstruktiver Chirurgie von der Lidplastik und Gesichtswiederherstellung über die Brustformung bis hin zur Nervenrekonstruktion oder motorischen Ersatzoperationen. Ein rechtzeitig durchgeführter chirurgischer Eingriff kann die Krankheitsentwicklung und Lebensqualität der Betroffenen entscheidend beeinflussen und im Fall einer Transformation zum malignen peripheren Nervenscheidentumor (MPNST) sogar lebensrettend sein.

## Abstract (en)

Neurofibromatosis type 1 (NF1, formerly Recklinghausen’s disease) is a genetic tumor predisposition syndrome in which the mutation of a tumor suppressor gene (*neurofibromin*) leads to the development of mostly benign neurofibromas of the skin and the central and peripheral nervous systems and malformations or tumors of other organ systems. Patients with NF1 should receive lifelong interdisciplinary care in specialized centers and important treatment decisions should be made by a regularly meeting interdisciplinary panel of experts. Plastic surgery plays an important role in the multidisciplinary management of all clinical forms of NF1-associated peripheral nerve sheath tumors, from cutaneous and subcutaneous to deep nodular and diffuse plexiform neurofibromas. Each patient requires individualized surgical planning, whereby the timing and extent of surgery are determined by the accompanying symptoms, functional and esthetic limitations, disease progression and potential malignant transformation. As any region of the body can be affected, the esthetic and reconstructive procedures required include a wide range of interventions, such as eyelid surgery and facial restoration to breast shaping and nerve reconstruction or motor replacement surgery. A timely surgical intervention can have a profoundly positive effect on the course of the disease and the quality of life of those affected and, in the case of transformation into a malignant peripheral nerve sheath tumor (MPNST), can even be lifesaving.

## Hintergrund

### Neurofibromatose Typ 1

Die Neurofibromatose Typ 1 (NF1) ist eine häufige autosomal-dominante Erkrankung (Häufigkeit bei 1 zu 2500 bis 3000 Lebendgeburten; [1]), welche durch eine Mutation des Neurofibromingens (NF1-Gen) auf Chromosom 17 verursacht wird. Dies führt zu übermäßigem Zellwachstum mit Tumorbildungen u. a. der Haut und des Nervensystems. Neben einer autosomal-dominant vererbten Form liegt in ca. 50 % der Fälle eine Spontanmutation vor.

Der frühere Namensgeber, Friedrich von Recklinghausen (1833–1910), hat als deutscher Pathologe diese krankhafte Ausbildung von Neurofibromen erstmals 1881 ausführlich beschrieben. Betroffene weisen typische Erscheinungsformen auf: Hellbraune Café-au-lait-Flecken, sommersprossenartige Flecken in der Achsel- und Leistenregion („Freckling“), Lisch-Knötchen in der Iris und kutan und tiefer liegende noduläre Neurofibrome, die meist erst ab der Pubertät auftreten, danach aber an Anzahl und Größe zunehmen und über 99 % aller Patient:innen betreffen [2]. Diese Neurofibrome bestehen hauptsächlich aus Schwann-Zellen, Fibroblasten und Mastzellen und können von jedem Nerv außerhalb von Gehirn oder Rückenmark ausgehen, jede Körperregion betreffen und verdrängend in normales Gewebe einwachsen. Neurofibrome können auch subkutan und im Verlauf der Nerven perlschnurartig vorkommen. Plexiforme Neurofibrome sind Geflechte von Nerventumoren, die von der Nervenscheide meist größerer Nerven ausgehen und unterschiedlich stark mit den Axonen verwachsen sind [3]. Das Entartungsrisiko für plexiforme Neurofibrome liegt bei ca. 10 %, wobei es dabei (über die Vorstufe atypischer Neurofibrome [„atypical neurofibromatous neoplasms of uncertain biologic potential“, ANNUBP]) zur Entwicklung sog. maligner peripherer Nervenscheidentumoren (MPNST) kommt, die eine sehr schlechte Prognose aufweisen [4].

Generell haben NF1-Patient:innen ein erhöhtes Risiko für Neoplasien (z. B. Mammakarzinom) im Vergleich zur restlichen Bevölkerung und auch dadurch eine Minderung der Lebenserwartung von ca. 10 Jahren, wobei im Vergleich zur nichtbetroffenen Bevölkerung insbesondere jüngere Erwachsene das höchste relative Risiko aufweisen. Häufig bilden sich auch im Kindesalter Sehbahngliome, welche in ca. 50 % symptomatisch sind. Ebenso treten bereits im Wachstumsalter knöcherne Fehlbildungen auf wie Keilbeindysplasien, Tibiadysplasien mit anterolateraler Verbiegung („bowing“) oder Tibiapseudoarthrose, Skoliosen. Aufgrund des progressiven Verlaufs der NF1 ist eine laufende medizinische Betreuung an einem spezialisierten Zentrum erforderlich (Pädiatrischer Vorsorgeplan NF1). Diese erfolgt in erster Linie durch Fachärzte der Pädiatrie, Neurologie, Onkologie, Augenheilkunde, diverse chirurgische Disziplinen (plastische Chirurgie, Neurochirurgie, Hals-Nasen-Ohren-Heilkunde, Viszeralchirurgie), Dermatologie, Radiologie bzw. Nuklearmedizin und medizinische Genetik.

### NF2-assoziierte Schwannomatose

Abzugrenzen ist die NF2-assoziierte Schwannomatose (früher Neurofibromatose Typ 2 oder kurz NF 2), die deutlich seltener auftritt (ca. 1:30.000) und häufiger Strukturen im zentralen Nervensystem (v. a. beidseitige Akustikusschwanomme, Gliome) betrifft [5]. Periphere Läsionen können auch hier auftreten (Schwannome). Zusätzlich kann es zu noch selteneren Formen der Schwannomatose kommen, denen andere Genveränderungen zugrunde liegen.

## Pathogenese

Neurofibrome infiltrieren, z. B. im Unterschied zu Schwannomen, diffus das Endoneurium entlang der Nervenfasern. Gehen sie von kleineren Nerven aus, breiten sie sich meist diffus im Peri- und Epineurium und auch im angrenzenden perineuralen Gewebe aus, während Neurofibrome mittelgroßer und großer Nerven in ihrer Ausdehnung oft zumindest weitgehend auf die Nerven begrenzt sind.

### Plexiforme Neurofibrome

Bei primär gutartigen plexiformen Neurofibromen (PN) sind nebeneinanderliegende Nervenfaszikel diffus von Neurofibromzellen infiltriert, die reichlich mukoide Matrix produzieren. Die befallenen Faszikel sind massiv aufgetrieben und verdickt („bag of worms“). Die PN bergen ein erhöhtes Entartungsrisiko [6, 7]. Etwa 30–50 % aller NF1-Patient:innen entwickeln ein solches PN, entweder subkutan oder an den Nervenstämmen. Diese entstehen in der Regel innerhalb der ersten Lebensjahre. Der natürliche Verlauf ist sehr unterschiedlich, sodass die Patient:innen regelmäßig untersucht werden sollten, um den Verlauf des individuellen Tumors abschätzen zu können [7].

#### Cave.

Plexiforme Neurofibrome weisen wie Neurofibrome größerer Nerven ein deutlich erhöhtes Entartungsrisiko auf als solitäre Neurofibrome der Haut.

## Diagnostik

### Klinisches Bild

Die überarbeiteten diagnostischen Kriterien für die NF1 sind Tab. 1 zu entnehmen.

**Tab. 1:** Überarbeitete diagnostische Kriterien der Neurofibrome Typ 1 [8]

Die Diagnose wird anhand klinischer Kriterien gestellt, wobei zwei oder mehr der angeführten Punkte erfüllt sein müssen
1. Sechs oder mehr Café-au-lait-Flecken größer als 5 mm im vorpubertären Alter, größer als 15 mm nach der Pubertät
2. Zwei oder mehr Neurofibrome bzw. ein plexiformes Neurofibrom
3. Freckling (sommersprossenartige Pigmentierung) im Achsel- bzw. Leistenbereich
4. Sehbahntumor
5. Zwei oder mehr Irishamartome (Lisch-Knötchen, Diagnostik mit Spaltlampe) oder mindestens zwei choroidale Abnormalitäten (helle, fleckenförmige Knötchen, Diagnostik mittels optischer Kohärenztomographie [OCT] bzw. Nahinfratrotspektroskopie [NIR])
6. Charakteristische Knochenveränderung (wie Keilbeinflügeldysplasie) oder Verbiegung langer Röhrenknochen mit oder ohne Pseudarthrose
7. Eine heterozygote, pathogene Variante des NF1-Gens mit variabler Allelfraktion von 50 % in sonst normalem Gewebe, z. B. weiße Blutkörperchen
8. Ein Verwandter 1. Grades (Elternteil, Geschwister, Kind) ist von NF1 gemäß diesen Kriterien betroffen

*NF1 *Neurofibrome Typ 1

### Bildgebung

#### Magnetresonanztomographie

Die Magnetresonanztomographie (MRT) ist richtungsweisend, um die beteiligten Nerven zu benennen und die Tumorausdehnung sowie Diffusionseigenschaften zu beurteilen [9, 10]. Neurofibrome imponieren tendenziell im MRT etwas inhomogen. Insbesondere an den Tumorpolen zeigt sich eine schlechtere Abgrenzbarkeit gegenüber den gesunden Faszikeln im Vergleich zu den Schwannomen. Zur näheren Differenzierung kommen hier vor allem diffusionsgewichtete Sequenzen („diffusion-weighted sequence“, DWI) zum Einsatz, um einen Hinweis auf die Dignität des Tumors zu bekommen. Mittels „apparent diffusion coefficient“ (ADC) wird das Maß der Diffusionsfähigkeit des Gewebes angegeben, wobei niedrige ADC-Werte auf eine eingeschränkte Diffusion hinweisen, was mit einem höheren Risiko für Malignität korreliert [9]. STIR-Sequenzen („short tau inversion recovery“) sind besonders geeignet, um die Tumorlast und das Tumorwachstum im Verlauf zu beurteilen [10].

Für die operative Planung sehr hilfreich ist die MR-Traktographie. Hierbei wird mittels diffusionsgewichteter Sequenzen der Verlauf der Nervenfasern dreidimensional rekonstruiert, um die Lagebeziehung und Abgrenzung zum Tumor darzustellen [11].

#### Nervenultraschall

Mittels hochauflösender Neurosonographie kann in der Operationsplanung bis auf Faszikelebene das Ausmaß der tumorbedingten Nervenverdrängung und -durchwachsung bestimmt und damit die chirurgische Entfernbarkeit abgeschätzt werden.

#### ^18^F-Fluorodesoxyglucose-Positronenemsissions‑/Magnetresonanz- oder Computertomographie

Die Bildgebung mittels ^18^F‑Fluorodesoxyglucose-Positronenemsissions‑/Magnetresonanz- oder Computertomographie (FDG-PET/MRT oder PET/CT) hat einen hohen Stellenwert in der Diagnostik, da die aktuelle Tumorlast und -aktivität beurteilt werden kann und eine hohe Sensitivität auch für asymptomatische entartete Tumoren besteht. Bei NF1-Patient:innen mit bekannten Tumoren oder mit erhöhtem Risiko für MPNST sollten diese regelmäßig mittels Bildgebung kontrolliert werden (MRT bzw. bei Hochrisikopatient:innen bzw. Patient:innen mit Verdacht auf maligne Transformation auch PET/MR oder ggf. PET/CT), um die aktuelle Tumorlast und -aktivität zu beurteilen [12].

**Abb. 1:**
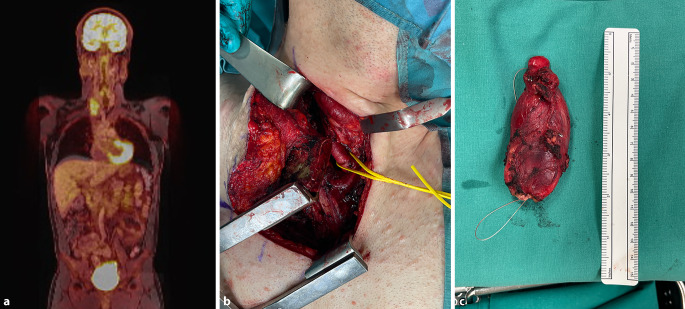
**a** Positronenemissionstomographie/Magnetresonanztomographie eines malignitätsverdächtigen Neurofibroms des N. vagus mit vermehrter Traceraufnahme im Bereich der rechten Thoraxapertur. **b** Zugang mittels oberer Osteotomie des Sternums nach Grünenfeld (L-förmige Osteotomie des Manubriums im Bereich des Sternoklavikulargelenks und der 1. Rippe). Der eingesetzte Wundspreizer ermöglicht den Zugang zwischen Sternum und Klavikula/1. Rippe. **c** Resezierter Tumor

In einer aktuellen Studie wurde gezeigt, dass der Wert für die maximale Traceraufnahmerate (SUV_max_ [„standard uptake value“]) bei malignen Tumoren im Rahmen der NF1 mindestens bei 3,15 lag [12]. Oberhalb dieses Werts muss eine Transformation befürchtet werden und die chirurgische Resektion sollte erwogen werden, wenngleich auch benigne Neurofibrome höhere SUV-Werte aufweisen können (Abb. 1).

## Behandlungsoptionen

### Konservative Therapie

In den letzten Jahren haben sich MEK-Inhibitoren (*M*AP/*E*RK-*K*inase 1/2, z. B. Selumetinib, Trametinib) als wirksame Medikamente gezeigt, um in chirurgisch schwierigen Fällen eine deutliche Größenreduktion des Tumors und Symptombesserung wie z. B. Schmerzlinderung zu erzielen [13–15]. Die European Medicines Agency (EMA) hat 2021 die Verwendung von Selumetinib für inoperable, symptomatische plexiforme Neurofibrome bei Kindern und Jugendlichen zwischen 3 und 18 Jahren zugelassen. Weitere MEK-Inhibitoren werden im Rahmen von Studien verwendet. Somit stellt die Operation weiterhin einen wichtigen Therapiebestandteil zur Behandlung der NF1-assoziierten PN dar, zumal MEK-Inhibitoren nicht ohne Nebenwirkungen sind und die PN nach Absetzen der Medikation beinahe regelhaft wieder symptomatisch werden und wachsen können.

Ob MEK-Inhibitoren neoadjuvant eingesetzt werden können, um damit eine Operabilität zu erreichen, wird man in Zukunft evaluieren müssen. MEK-Inhibitoren können eine maligne Transformation (s. unten) jedoch nicht verhindern.

### Chirurgische Therapie

#### Indikation zur chirurgischen Behandlung

Grundsätzlich sollten Patient:innenbetreuung und Therapieentscheidungen an Zentren mit NF1-Expertise und im interdisziplinären Austausch stattfinden, insbesondere sollte jede Entscheidung bez. der richtigen Therapieindikation bei peripheren Nervenscheidentumoren und hinsichtlich chirurgischer und nichtchirurgischer Optionen gemeinsam diskutiert und entschieden werden.

Die chirurgische Indikation ergibt sich bei Schmerzen, Funktionsdefiziten und ästhetischen Deformitäten mit erheblichem Einfluss auf Alltagsaktivität, soziale Beziehungen und Lebensqualität der Betroffenen und bei Verdacht auf maligne Entartung.

#### Maligne periphere Nervenscheidentumoren

Patient:innen mit großer Gendeletion, multiplen plexiformen Neurofibromen und MPNST in der (Familien‑)Anamnese haben ein erhöhtes Risiko für MPNST. Diese können in jedem Alter auftreten, jedoch sind oft junge Erwachsene betroffen (medianes Alter bei 30 Jahren). Insbesondere in tieferen plexiformen Läsionen können sich maligne periphere Nervenscheidentumoren mit oft infauster Prognose entwickeln, die mit rechtzeitiger Exzision deutlich verbessert werden kann.

Hinweise für eine mögliche maligne Entartung sind:

- neu aufgetretene bzw. anhaltende Schmerzen,
- neu auftretende neurologische Ausfallerscheinungen,
- schnelles Tumorwachstum,
- Bildung eines soliden Knotens,
- Zeichen für Malignität in der Bildgebung (MRT oder FDG-PET).

Die Indikation zur chirurgischen Resektion sollte in diesen Fällen großzügig gestellt werden, insbesondere beim Zutreffen von mehr als einem der oben genannten Indizien.

#### Chirurgische Techniken abhängig von Tumorart und -lokalisation

Das operative Vorgehen kann nach folgenden Tumormanifestationen der peripheren Nerven bei NF1 unterschieden werden:

1. kutane Neurofibrome,
2. subkutane/tiefer gelegene Neurofibrome.

Generell können alle Neurofibrome nach der Wachstumsart in nodulär, diffus oder plexiform eingeteilt werden [16]. Kutane Neurofibrome werden des Weiteren in latent, flach, sessil, globulär, pedunkulär eingeteilt [17].

##### Kutane Neurofibrome.

Oberflächliche, kutane Neurofibrome sind in der Regel gutartig und können exzidiert oder mittels Laser (CO_2_- oder Erbium:YAG-Laser) oder Radiofrequenzablation abgetragen werden [18, 19]. Weitere Methoden zur Tumorablation stehen mit der Elektrodissektion zur Verfügung, wobei die Tumoren mittels monopolarer Diathermie und feinem Nadelaufsatz koaguliert werden [16, 20]. Beim „high intensity focused ultrasound“ (HIFU) werden die Tumoren gezielt erhitzt, ohne die Hautbarriere vollständig zu durchbrechen. Die Effektivität und Sicherheit in dieser Indikation wird aktuell untersucht (NCT05119582).

Kutane Neurofibrome stellen für die Betroffenen vor allem ein häufig schwer belastendes ästhetisches Problem dar, wenn die Papeln in großer Anzahl über den ganzen Körper verteilt auftreten. In manchen Fällen kommen Juckreiz und Schmerzen durch mechanische Hautreizung (z. B. Gürtelbereich, Fuß oder Knöchel) hinzu. Bei diesen kutanen Neurofibromen ist die Haut von Tumorzellen durchsetzt und ausgedünnt, charakteristisch sind auch Hyper- oder Hypopigmentierung, Ulzeration sowie gelegentlich Juckreiz. Diese Tumoren gehen meist von kleinen Hautästen aus, die ihre Funktion entweder schon verloren haben oder deren Verlust sich klinisch nicht auswirkt. Sie treten flächig disseminiert am ganzen Körper auf und führen zu bizarren Entstellungen (v. a. an Extremitäten, Rumpf, Kopf, Orbita).

##### Subkutane/tiefer gelegene Neurofibrome.

Bei ausgedehnten subkutanen Neurofibromen hat sich der Tumor in der Regel diffus verbreitet und die Abgrenzung zu einem bestimmten Nerven ist selten möglich. Die Resektion erfolgt hier großzügig und erfordert manchmal die Mitnahme der darüber liegenden Haut. Die Resektion dieser Läsionen kann mit erhöhter Blutungsneigung vergesellschaftet sein und diese sollte im perioperativen Management bedacht werden.

Da die meisten Tumoren bei NF1 gutartig sind, sind serielle Exzisionen und Rekonstruktion mit umliegendem Gewebe (Primärverschluss oder lokale Lappenplastiken) bei symptomatischen Patient:innen sinnvoll, auch wenn das Risiko eines Lokalrezidivs besteht. Eine radikale Tumorexzision ist indiziert, wenn Hinweise auf Malignität bei der klinischen Untersuchung (z. B. anhaltende Schmerzen, schnelles Wachstum) oder eine entsprechende Bildgebung vorliegen.

**Abb. 2:**
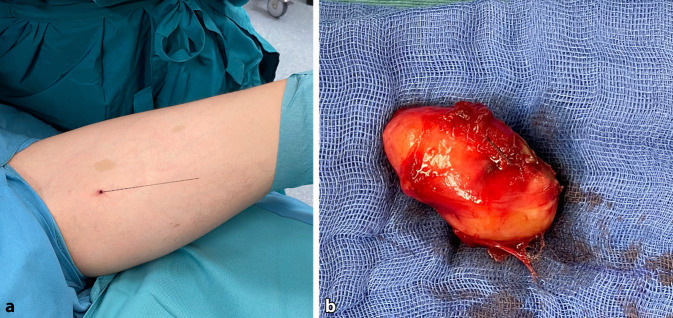
Präoperative Drahtmarkierung (**a**) des zu resezierenden Tumors (**b**)

Vor dem Eingriff muss der Chirurg anhand klinischer und radiologischer Untersuchungen abschätzen, ob und in welchem Ausmaß durch die Resektion eine funktionelle Beeinträchtigung zu erwarten ist und wie diese rekonstruiert werden kann. Beim Vorliegen multipler Tumorverbände kann eine präoperative Drahtmarkierung des zu resezierenden Tumors hilfreich sein (Abb. 2).

**Abb. 3:**
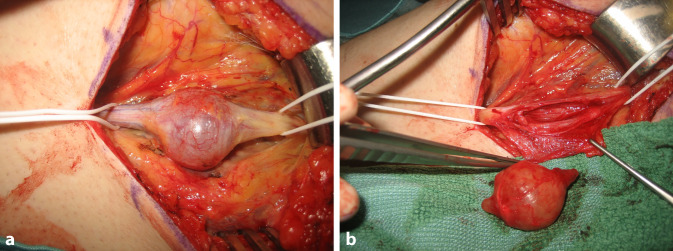
Interfaszikuläre Exstirpation eines plexiformen Neurofibroms am N. peroneus communis bei motorischer Schwäche (**a**). Eine Rekonstruktion ist aufgrund des Faszikelerhalts bei guter Tumorabgrenzbarkeit nicht erforderlich (**b**)

**Abb. 4:**
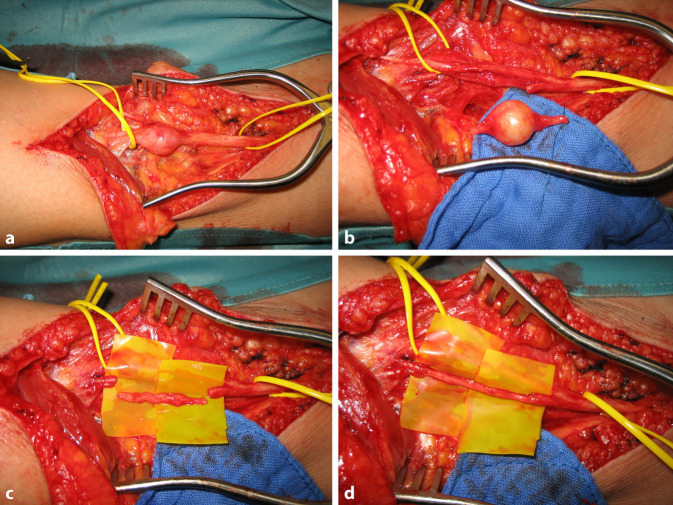
Partielle Rekonstruktion des N. tibialis nach Resektion eines Neurofibroms mit faszikulärer Beteiligung: Neurofibrom des N. tibialis (**a**). Resektion unter Mitnahme eines faszikulären Stranges (**b**). Partielle Nervenrekonstruktion mittels N. suralis-Interponat (**c**, **d**)

Oft sind die klinischen Symptome durch eine Verdrängung und Kompression der Axone durch den Tumor bedingt, welche nach nervenschonender Tumorexstirpation dekomprimiert und entlastet werden. Bei klassischen plexiformen Neurofibromen, welche oft einem großen Nerven entspringen, wird dieser unter Lupenvergrößerung präpariert und von dem restlichen Nerven getrennt. Diese Tumoren sind eine mikrochirurgische Herausforderung, da die Nervenhauptstämme nicht selten langstreckig und mit Beteiligung aller Faszikel infiltriert sind, manchmal sind sogar mehrere Nerven gleichzeitig befallen. Wenn sie im Stammbereich (z. B. intraabdominal, intrathorakal oder intrapelvin) entstehen, werden sie erst symptomatisch und entdeckt, wenn eine beträchtliche Ausdehnung erreicht ist [21]. Mittels intraneuraler Dissektion und faszikelsparender Enukleation soll nur der betroffene Nervenanteil mit seinem „feeding fascicle“ reseziert werden. Beginnend an den Tumorpolen wird der Tumor vom betroffenen Nerven unter laufender Nervenstimulation abpräpariert, da direkt im Tumor die funktionstragenden Faszikel schlechter erkannt werden können [22, 23]. Mit entsprechender Erfahrung gelingt dies oft ohne wesentliche motorisch-sensible Ausfälle (Abb. 3). Manchmal ist es nicht möglich, funktionserhaltend zu operieren, sodass in gleicher Sitzung eine Rekonstruktion (z. B. durch Nerveninterponat, Nerven- oder Sehnentransfer) erforderlich werden kann (Abb. 4). Zudem ist wegen der unklaren Tumorgrenzen eine vollständige Exstirpation nicht immer möglich. Ein weiteres Problem ist die lakunenartige Veränderung der Gefäße ohne eigentliche Gefäßwand bei Hypervaskularität der Tumoren, sodass die Blutstillung schwierig und der Blutverlust vermehrt sein kann [24].

Die chirurgischen Optionen müssen kritisch abgewogen werden, da leider nicht immer ein voll befriedigendes Resultat erzielt werden kann (Biopsie vs. radikale Resektion mit Funktionsverlust, mit oder ohne Nervenrekonstruktion). Die genannten Faktoren müssen präoperativ klar und deutlich angesprochen werden.

Ausgedehnte plexiforme Neurofibrome entarten am häufigsten zu einem MPNST (Risiko bei 5–10 %; [24, 25]). Hochrisikopatient:innen sind v. a. Menschen mit NF1 und großen Gendeletionen, multiplen plexiformen Neurofibromen oder einer persönlichen Vorgeschichte eines MPNST bzw. einer positiven Familienanamnese. Wenngleich MPNST in jedem Lebensalter auftreten können, so liegt das mediane Alter bei 30 Jahren, d. h. v. a. junge Patient:innen sind betroffen. Beim Hinweis auf eine maligne Entartung sollte die chirurgische Resektion großzügig indiziert werden.

#### Chirurgisches Vorgehen abhängig von anatomischer Region

##### Kopf-Hals-Bereich, orbitotemporale Läsionen und hemifaziale Hypertrophie.

Das Spektrum kraniofazialer Weichteilanomalien bei NF1 ist sehr breit gefächert und betrifft die gesamte Hals-Gesichts-Region (Orbita, Augenlider, temporoaurikuläre Region, Carotisloge, Nacken etc.; [26–28]).

Die chirurgische Behandlung ist aufgrund der vielen beteiligten Strukturen sehr vielfältig und anspruchsvoll. Primäre Ziele sind eine Schmerzreduktion und Korrektur von Funktionsdefiziten (z. B. Sehstörungen, Probleme beim Kauen, Atembeschwerden aufgrund von Neurofibromen), aber auch eine verbesserte Ästhetik durch Beseitigung der stigmatisierenden Hautüberhänge ist erstrebenswert, da diese für die Betroffenen gerade im Gesichtsbereich aus psychosozialen Gründen eine enorme Belastung darstellen. Die chirurgischen Techniken reichen von der Laserablation zahlreicher kleiner Hautpapeln bis zur allogenen Gesichts(teil)transplantation bei extremen Deformitäten durch herabhängende Tumormassen [29, 30].

Einseitige orbitotemporale Läsionen werden auch als „hemifaziale Hypertrophie“ (früher „Elephantiasis neuromatosa“) bezeichnet und führen durch eine einseitige diffuse Proliferation von Schwann-Zellen und Axonen zu einer massiven, entstellenden Gewebevermehrung, teilweise mit einer knöchernen Deformität. Chirurgische Richtlinien für diese speziellen Tumorbildungen existieren ebenso wie operative Konzepte für die knöcherne und die palpebrale Beteiligung [31, 32].

**Abb. 5:**
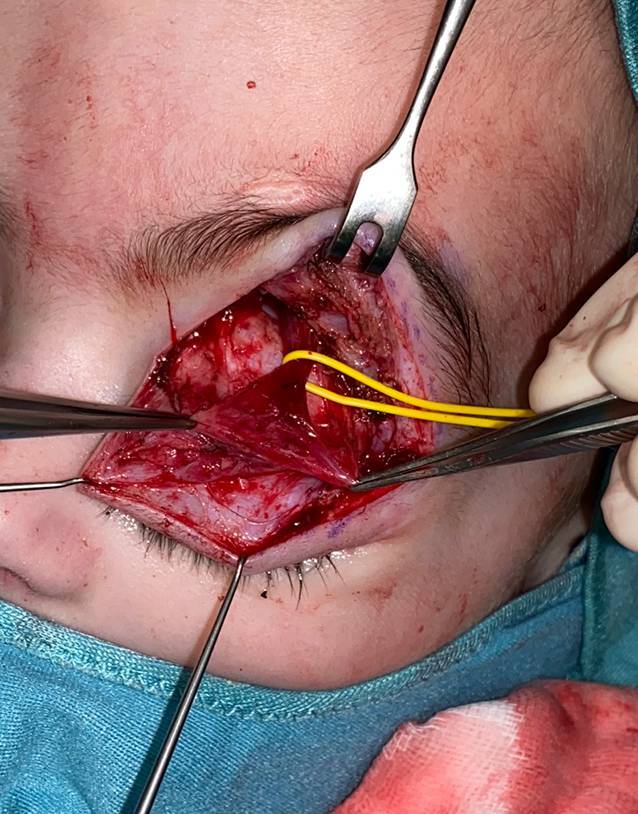
Tumorreduktion orbital links unter Schonung des M. levator palpebrae superioris. Die Verdrängung durch die Tumormassen hat zu einer chronischen Überdehnung des Muskels mit insuffizienter Lidhebung geführt, weshalb im selben Eingriff eine Levatorraffung durchgeführt wurde

Typische Eingriffe im Gesichtsbereich umfassen:

1. En-bloc-Exzisionen nach den Prinzipien der ästhetischen Gesichtseinheiten
2. Aufhängeplastiken/Suspensionen herabhängender Gesichtspartien, z. B. von
  oberem, mittlerem (inkl. des malaren Fettpolsters) und/oder unterem (inkl. der Mundpartie) Gesichtsdrittel
  Augenbrauen und/oder Ohren mit autologer Fascia lata oder nicht resorbierbarem Nahtmaterial (Nylon oder Stahldraht)
3. Lidplastiken, laterale Kanthopexie und horizontale Verkürzung des Tarsus
  Die Oberlidplastik erfolgt bevorzugt mit transversaler elliptischer Exzision mit sorgfältiger Präparation zum Erhalt der Lidhebung (M. levator palpebrae superioris, M. tarsalis superior) und des Tarsus (Abb. 5)
4. Stirnlappenplastik zur Nasenrekonstruktion [27, 31]

Wichtige chirurgische Prinzipien bei diesen Gesichtstumoren sind:

- Zur möglichst schonenden Tumorexzision sind verschiedenste chirurgische Zugänge (bikoronal, semikoronal, subziliär, intraoral, präaurikulär, retroaurikulär und Facelift) notwendig.
- Die Äste des N. trigeminus und des N. facialis sollten geschont werden, außer bei massiven plexiformen Neurofibromen, die keine Kapseln haben und in das angrenzende Gewebe infiltrieren.
- Das primäre Ziel ist oft nicht die radikale Exzision, sondern nur eine subtotale Exzision zur Tumorreduktion mit konsequenter funktioneller und ästhetischer Verbesserung.
- Bei sehr großen plexiformen Neurofibromen dienen die ästhetischen Einheiten der weniger (oder nicht) beeinträchtigten Gegenseite als Orientierung zur Festlegung des Umfangs der Gewebeexzision.
- Um länger eine stabile Repositionierung ptotischen Gesichtsgewebes zu erreichen, sollte dieses mit nichtresorbierbarem Nahtmaterial und Faszienzügeln an knöchernen Strukturen verankert werden (z. B. Jochbeinbogen, lateraler Orbitarand, Os temporale). Hierbei ist eine initiale Überkorrektur sinnvoll, da eine langzeitstabile Aufhängung die Auswirkungen der Schwerkraft auf die Ausdehnung des Tumorgewebes verhindern oder abschwächen kann.

##### Äußeres Ohr.

**Abb. 6:**
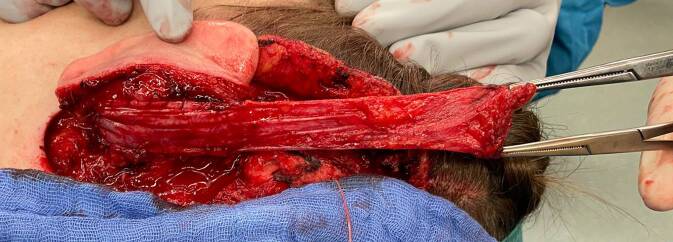
Aufhängeplastik mit Fascia-lata-Streifen nach Tumorreduktion bei herabgesenktem linkem Ohr. Kranial wird die Faszie mittels Knochenanker transossär fixiert

Bei Tumorinfiltration des äußeren Ohrs kann aus funktionellen und ästhetischen Gründen ein chirurgisches Vorgehen indiziert sein: Einerseits, wenn durch Verlegung des äußeren Gehörgangs durch Tumorgewebe das Hörvermögen gestört ist, andererseits aus kosmetischen Gründen, wenn die äußere Architektur stark verzerrt und das Ohr abgesackt ist. Ziel des chirurgischen Eingriffs ist die möglichst vollständige Exzision des Tumors unter Erhalt oder die Wiederherstellung der äußeren Ohrkontur. Aufgrund des postoperativen Weichteilverlustes kann eine Ohraufhängeplastik (mit Fascia-lata-Streifen und knöcherner Fixation) sinnvoll sein (Abb. 6; [33]).

##### Hals.

Das Spektrum der NF1-assoziierten Tumoren reicht hier von kleinen asymptomatischen Läsionen mit wenig oder keinem Wachstum (die nur beobachtet werden) bis hin zu Raumforderungen mit Verdrängung von Atemwegen und Verdauungstrakt, Nervenkompression und schweren ästhetische Entstellungen. Neurofibrome ausgehend vom N. vagus können sich im Extremfall von der Schädelbasis bis zum Mediastinum erstrecken und aufgrund ihrer Nähe zu Rachen, Luft- und Speiseröhre schwere Atemwegs- und Schluckbeschwerden verursachen. Von Nervenwurzeln des Plexus cervicalis entspringende Neurofibrome können die Atemwege ebenso komprimieren und zu neurologischen Ausfällen des Plexus brachialis führen, selten sogar die großen Halsgefäße komprimieren oder das Wachstum der Halswirbelsäule beeinträchtigen. Die präoperative Planung mit gezielter Bildgebung (MRT und/oder CT) ist obligat [34].

##### Wichtig.

Aufgrund der komplexen Präparation und der zahlreichen vitalen Strukturen auf engstem Raum sollten Resektionen tief liegender oder großer Tumoren am Hals multidisziplinär (plastische Chirurgie, Hals-Nasen-Ohren-Heilkunde [HNO], Neurochirurgie, Allgemein- und Kinderchirurgie) geplant und durchgeführt werden.

##### Oberer Thorax (Mediastinum).

Mediastinale Tumoren sind nicht selten nur schwer zugänglich und können eine Klavikulaosteotomie mit Abtrennung des Musculus pectoralis major erfordern oder eine Disartikulation des Sternoklavikulargelenks, selten sogar eine Sternotomie. Bei beidseitiger retrosternaler Beteiligung sollten die Tumorentfernung möglichst in einer Operation versucht werden, um eine erneute Sternotomie zu vermeiden. Auch wenn Resektionen der Thoraxneurofibromatose oft weniger blutig als im Gesichtsbereich sind, sollten Bluttransfusionen und eine postoperative Versorgung auf der Intensivstation bedacht werden [35].

##### Brust und Axilla.

Besonders an der Brust und in der Axilla entsteht nach Tumorresektion ein Weichteildefizit, welches zur Wiederherstellung der Symmetrie im selben Eingriff mittels Implantat oder gestieltem Lappen ausgeglichen werden muss. Da das Mammakarzinomrisiko bei NF1-Patientinnen deutlich erhöht ist (> 5-fach), gehört zum plastisch-chirurgischen Behandlungsspektrum auch die Brustrekonstruktion mit Eigengewebe oder Implantaten nach Ablation [36].

##### Plexus brachialis.

Neurofibrome verursachen durch Kompression des Armnervengeflechts oft starke Schmerzen, meist bevor sensible oder motorische Funktionsausfälle auftreten. Da die Tumoren oft nicht direkt von Nerven des Plexus brachialis ausgehen, können die Symptome durch Tumorexstirpation verringert oder komplett geheilt werden. Dem hochauflösenden Nervenultraschall („high resolution nerve ultrasound“, HRNUS) kommt hier wichtige Bedeutung bei Diagnostik und Screening zu. Die genaue Lokalisation des Tumors und Abgrenzung zu den faszikulären Strukturen lassen sich für die Operationsplanung mittels Traktographie im MRT darstellen. Bei Tumoren im Bereich der oberen Thoraxapertur (Nervenwurzeln C8/Th1) kann es notwendig sein, eine obere Sternotomie durchzuführen, um ausreichend Zugang zum Tumor zu bekommen (siehe oben). Durch die intraoperative Nervenstimulation lässt sich testen, ob ein funktioneller Schaden nach der Tumorexstirpation zu erwarten ist. Die funktionelle Nervenrekonstruktion ist jedoch nur in Ausnahmefällen erforderlich.

##### Wichtig.

Die Präparation plexusnaher Neurofibrome erfordert mikrochirurgische Ausrüstung sowie eine intraoperative Nervenüberwachung, die Tumoren sind oft stark vaskularisiert und ein großer Blutverlust kann die Operationen komplizieren.

##### Plexus lumbosacralis und tiefer Beckenbereich.

**Abb. 7:**
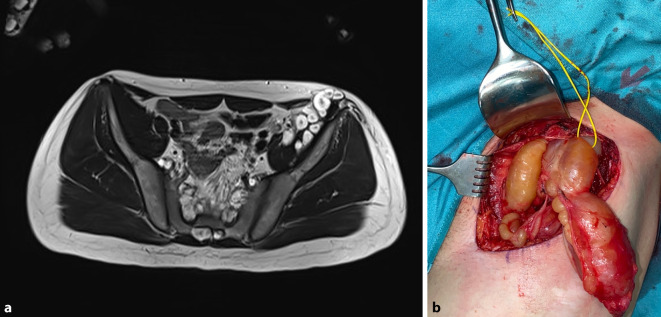
**a** Ausgeprägte Tumormassen im Bereich der linken Leiste. **b** Die perlschnurartigen Formationen werden reseziert, um den N. cutaneus femoris lateralis zu entlasten

Neurofibrome in diesem Bereich (retroperitoneal) sind aufgrund der tiefen Lage und ihrer Nachbarschaft zu neurovaskulären Strukturen und inneren Organen oft schwer zu erreichen. Die Tumoren können jedoch auch oberflächlich auftreten und bei verdrängendem Wachstum Nervenkompressionssyndrome an typischen Stellen auslösen, z. B. Kompression des N. cutaneus femoris lateralis (Abb. 7). Die Indikation bei tief, z. B. retroperitoneal, gelegenen Tumoren ist besonders kritisch zu überprüfen, da eine operative Resektion hohe Risiken birgt. Hierbei muss abgewogen werden, ob eine Biopsie und ein Debulking sinnvoller ist als eine ohnehin kaum mögliche radikale Tumorentfernung. Zudem sind die rekonstruktiven Möglichkeiten aufgrund der schwer erreichbaren Lokalisation begrenzt. Das operative Vorgehen sollte mit anderen chirurgischen Disziplinen (Viszeralchirurgie, Neurochirurgie) geplant werden.

#### Chirurgische Besonderheiten bei NF1

##### Erhöhtes Blutungsrisiko.

Neurofibrome sind nicht eingekapselte Tumoren mit unauffälligen, dünnwandigen Gefäßen. Sie sind häufig mit anormalen Versorgungsgefäßen verbunden, die zu Blutungen aus brüchigem fibromyxomatösem Gewebe führen können [37, 38]. Es besteht ein erhöhtes Risiko perioperativer und postoperativer Blutungen, die ein angepasstes Vorgehen erfordern:

- kontrollierte Hypotonie durch die Anästhesie,
- präoperative Tumeszenzinfiltration (0,9 % NaCl-Lösung mit Adrenalinzusatz),
- Fibrinversiegelung, bipolare Schere,
- schonende Präparation mit bipolarer Koagulation, sorgfältige Gefäßligatur,
- routinemäßige Einlage von Redon-Drainagen,
- evtl. präoperative Eigenblutspende.

##### Hautqualität.

Die Haut bei Patient:innen mit NF1 zeigt häufig fehlende Elastizität mit der Tendenz zu fortschreitender Hautinfiltration und Rezidiven mit erhöhtem Gewebevolumen und der daraus resultierenden Schwerkraftwirkung. Dies kann eine fortschreitende Verschlechterung der funktionellen und ästhetischen Ergebnisse bewirken, sodass immer wieder chirurgische Nachbesserungen notwendig werden können. Eine dauerhafte und stabile Suspension kann theoretisch die Auswirkungen der Schwerkraft auf die Ausdehnung des Tumorgewebes verhindern oder abmildern. Zudem ist die Dermis brüchig und reißt während der Operation leicht ein. Diese Eigenschaft der Haut spielt insbesondere eine Rolle, wenn an Körperteilen operiert wird, bei denen die Kontur äußerst wichtig ist, wie z. B. den Ohren, damit diese erhalten bleibt.

##### Interdisziplinäres operatives Vorgehen.

Durch das ubiquitäre Vorkommen der Nerventumoren ist nicht selten ein interdisziplinäres operatives Vorgehen notwendig. Typische Beispiele für Kooperationspartner der Plastischen Chirurgie sind:

- Neurochirurgie: Beteiligung proximaler Nervenwurzeln im Bereich des Neuroforamens und Spinalkanals
- Viszeral- und Thoraxchirurgie: Neurofibrome im Retroperitoneum (Plexus lumbalis) oder pleuranahe an der Brustwand
- HNO- und Thoraxchirurgie: Tumoren im tiefen Halsbereich und Mediastinum

### Behandlung in Spezialzentren

Aufgrund ihrer erhöhten Tumordisposition sollten Patient:innen mit NF1 lebenslang in spezialisierten Zentren interdisziplinär betreut werden. Ein multiprofessionelles Team aus Ärzt:innen, Pflegepersonen, Therapeut:innen (Ergo‑, Physio- und Logopädie) und psychosozialem Team ([Neuro-]Psychologie, Sozialarbeit, Pädagogik) sollte zur umfassenden Betreuung von Betroffenen und deren Familien unbedingt zur Verfügung stehen. Regelmäßige Expertentreffen (NF-Board) ermöglichen gezielte Falldiskussionen, Verlaufsbeobachtungen und Therapieplanungen unter Beteiligung verschiedenster Fachrichtungen (z. B. Pädiatrie, Onkologie, Ophthalmologie, Radiologie, Neurochirurgie, HNO, plastische Chirurgie, Orthopädie, Endokrinologie u. a.).

An der Universitätsklinik für Kinder- und Jugendheilkunde der Medizinischen Universität Wien (im AKH Wien) wurde zusammen mit der Patientenorganisation „NF Kinder“ das NF-Kinder Expertenzentrum für Neurofibromatose etabliert.

- **NF Kinder Expertisezentrum**
  - Abteilung für Neonatologie, Pädiatrische Intensivmedizin und Neuropädiatrie
  - Univ. Klinik für Kinder- und Jugendheilkunde
  - Universitätsklinikum Allgemeines Krankenhaus Wien
  - E‑Mail: NFKinderExpertisezentrum@akhwien.at
  - Tel. +43 (0)1 40400 31780 (Mo.–Fr. 13–15 Uhr)

#### Patientenorganisation

NF Kinder (für Betroffene jeden Alters!) hilft bei der Suche nach medizinischen Ansprechpartnern und bietet ein vielfältiges Unterstützungs- und Informationsangebot an.

- **NF Kinder – Hilfe für Neurofibromatose-Patient:innen und Angehörige, Österreich**
  - Büroadresse: Servitengasse 5/16, 1090 Wien
  - Web: www.nfkinder.at
  - E‑Mail: kontakt@nfkinder.at
  - Tel. +43 699 16624548

## Schlussfolgerung

Neurofibromatose 1 (NF 1) ist eine häufige genetische Erkrankung, die am gesamten Körper zu oberflächlichen kutanen und subkutanen, tiefer gelegenen und diffusen plexiformen Tumorbildungen (Neurofibromen) führen kann. Diese Neurofibrome, bestehend hauptsächlich aus Schwann-Zellen, Fibroblasten und Mastzellen, können von jedem Nerv außerhalb von Gehirn oder Rückenmark ausgehen, jede Körperregion betreffen und in normales Gewebe eindringen und dieses verdrängen. Plexiforme Neurofibrome sind subkutan oder tiefer gelegene Tumoren, welche von der Nervenscheide meist größerer Nerven ausgehen, unterschiedlich stark mit den Axonen verwachsen sind und in ca. 10 % der Fälle zu malignen peripheren Nervenscheidentumoren (MPNST) entarten, die eine sehr schlechte Prognose zeigen. Patient:innen mit NF 1 sollten aufgrund dieser erhöhten Tumordisposition lebenslang in spezialisierten Zentren interdisziplinär betreut, beraten und behandelt werden.

Die plastische Chirurgie spielt in der multidisziplinären Behandlung aller klinischen Formen der NF1 eine wichtige Rolle, die Anforderungen sind komplex und erfordern ein breites Spektrum sowohl an rekonstruktiven als auch ästhetisch-chirurgischen Techniken sowie die gesamte periphere Nervenchirurgie. Zeitpunkt und Ausmaß der Operationen wird von Symptomatik, funktionellen und ästhetischen Einschränkungen, Fortschreiten der Krankheit und potenzieller maligner Entartung bestimmt. Die Exzision von Neurofibromen kann aufgrund ihrer Größe, Lokalisation und Hypervaskularität kompliziert sein. Besonders schwierig und anspruchsvoll ist die Tumorexzision und Defektrekonstruktion im Gesichts- und Halsbereich. Ein rechtzeitig durchgeführter chirurgischer Eingriff kann die Krankheitsentwicklung und Lebensqualität der Betroffenen äußerst positiv beeinflussen und im Fall eines malignen peripheren Nervenscheidentumors (MPNST) sogar lebensrettend sein.
